# From Soil to Wine: Influence of Vegetative Covers on Microbial Communities and Fermentative Dynamics in *Cabernet Sauvignon*

**DOI:** 10.3390/microorganisms13122804

**Published:** 2025-12-09

**Authors:** Gerardo Leal, Joan Miquel Canals, Gemma Beltran, Álvaro Peña-Neira, Carla Jara, Jaime Romero, Carolina Ramírez, René Sanz

**Affiliations:** 1 Departament de Bioquímica i Biotecnologia, Facultat d’Enologia de Tarragona, Universitat Rovira i Virgili, C/Marcel.li Domingo 1, 43007 Tarragona, Spain; gerardo.leal@estudiants.urv.cat (G.L.); jmcanals@urv.cat (J.M.C.); gemma.beltran@urv.cat (G.B.); 2 Departamento de Agroindustria y Enología, Facultad de Ciencias Agronómicas, Universidad de Chile, Santa Rosa 11350, Santiago 7830489, Chile; apena@uchile.cl; 3 Laboratorio de Biotecnología de Alimentos, Instituto de Nutrición y Tecnología de los Alimentos (INTA), Universidad de Chile, El Líbano 5524, Santiago 7830489, Chile; carolina.ramirez.saavedra@gmail.com; 4 Departamento de Investigación y Desarrollo, Viña Santa Rita, Camino Padre Hurtado 0695, Alto Jahuel, Buin 8081460, Chile; rsanz@santarita.cl

**Keywords:** vegetative covers, *Cabernet Sauvignon*, microbiota, wine, Chile, fermentation

## Abstract

The implementation of vegetative cover crops in vineyards is a sustainable alternative to chemical weed control, potentially influencing both soil fertility and grape-associated microbiota. This study evaluated the impact of six groundcover management strategies under vines—white clover (*Trifolium repens*), red clover (*Trifolium pratense*), burr medic (*Medicago polymorpha*), lupine (*Lupinus albus*), spontaneous weeds, and an herbicide-treated control—on the microbial dynamics and physicochemical properties of *Cabernet Sauvignon* must and wine from the Maipo Valley, Chile. Amplicon sequencing of bacterial (16S rRNA) and fungal (ITS) communities was combined with spontaneous fermentation trials and chemical analyses of must and wine. Fungal and bacterial communities on grape surfaces were dominated by *Ascomycota* and *Proteobacteria*, respectively, with no significant compositional differences among treatments. During fermentation, *Metschnikowia* and *Tatumella* were the most abundant non-*Saccharomyces* and bacterial genera, respectively, showing dynamic shifts across fermentation stages. Legume-based covers, particularly red clover, increased wine total acidity and polyphenol index while reducing pH. Correlation analyses revealed associations between specific microbial taxa (*Metschnikowia*, *Cohnella*, *Saliterribacillus*) and key enological parameters. Overall, these findings demonstrate that leguminous cover crops subtly modulate vineyard microbial ecology and fermentation outcomes, offering an environmentally sustainable pathway to enhance enological differentiation in semi-arid viticultural regions.

## 1. Introduction

The microbiota on the surface of grape berries is composed of native yeasts and bacteria, which play a crucial role in fermentation processes and can affect the final wine sensory profile [1,2]. This community is very sensitive to various environmental factors such as rain, temperature and agronomic strategies applied in the vineyard, which influence the composition and abundance of yeasts and bacteria on grape berries [3,4].

Traditionally, the vineyard management has depended on the use of agrochemicals to eliminate weeds, in this way, to optimize crop yields. However, this approach has elevated growing concerns regarding its environmental impact, the loss of microbial biodiversity, and soil degradation [5]. However, studies have reported that native microbiota of grape berries does not demonstrate significant variations under natural soil coverage. This suggests that factors such as microclimate, biological vectors and foliar practices may have a greater influence [6,7]. Despite this, the use of agrochemicals is associated with a reduction in the diversity of yeasts and bacteria on the berries, which may affect spontaneous fermentation processes and the final sensory profile of the wine [8,9].

In this context, the implementation of cover crop represents a sustainable alternative that reduces the need for chemical inputs, promotes biological activity in the vineyard, improves soil structure, increases natural weed control, and supports the development of a microbiota on the berry surface [10]. Studies by [11,12] have shown that inter-row cover crops in vineyards can modify the microbial diversity of the berries by varying the microclimate and canopy relative humidity. Additionally, geographic location and terroir influence microbial composition, creating a microbial fingerprint that can be traceable to their origin and attend as enological differentiation markers [13,14]. Moreover, Refs. [15,16] reported that the use of *Phacelia tanacetifolia* or *Secale cereale* as cover crop enhances microbial diversity on *Cabernet Sauvignon* berries during ripening.

In Chile, the most widely produced wines come from the *Cabernet Sauvignon* cultivar. These vineyards are primarily located in the Maipo Valley, which offers ideal agroclimatic conditions for enological expression and high-quality wine production [17]. The use of cover crops in vineyards is a sustainable soil management strategy widely adopted in the world’s leading wine regions [18,19,20]. However, most existing studies focus on the effects of cover crops on soil nutrients and fruit quality, with relatively few addressing their impact on the microbial diversity of grape berries.

In this study, we evaluated the impact of different inter-row vineyard management strategies on the surface microbiota of *Cabernet Sauvignon* grapes in the Maipo Valley. The treatments included white clover (*Trifolium repens*), red clover (*Trifolium pratense*), burr medic (*Medicago polymorpha* L.), lupine (*Lupinus albus*), natural weed cover, and a conventional herbicide-treated control. These legumes were selected for their ability to fix nitrogen, improve soil fertility, and enhance biodiversity. They also help maintain vineyard ecological balance, control vine vigor, and improve grape quality in sustainable systems [11,20]. To investigate the microbiological implications of these practices, amplicon sequencing of bacterial and fungal communities was conducted to assess differences in microbial diversity and composition across treatments. In addition, fermentation trials using grapes from each treatment evaluated whether shifts in epiphytic microbiota could influence spontaneous fermentation dynamics and modulate the sensory attributes of the resulting wines. Together, these approaches provide new insights into how sustainable groundcover strategies shape the microbial terroir of vineyards and create opportunities for enological differentiation.

## 2. Materials and Methods

### 2.1. Study Site and Cultivar

**Figure 1:**
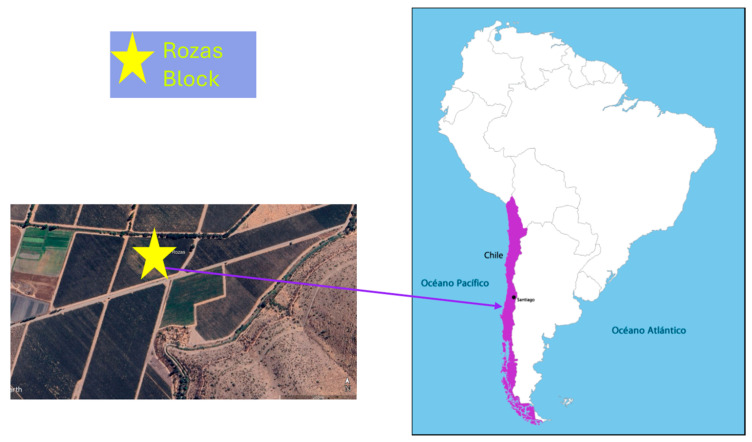
Geographical location of the study site (Rozas Block) within the experimental vineyard situated in the Metropolitan Region of Chile (33°43′7.28″ S; 70°39′53.23″ W).

The study was conducted in a vineyard located in Alto Jahuel, Maipo Valley (Rozas block, latitude 33°43′7.28″ S and longitude 70°39′53.23″ W, (Figure 1), characterized by a Mediterranean semi-arid climate with low annual precipitation (approximately 300–400 mm), concentrated mainly in winter (June–August). The soils are of alluvial origin, with gravelly and rocky subsoils. The experimental cultivar was *Cabernet Sauvignon* (*Vitis vinifera* L.) clone C46, own rooted plants established in 2015.

### 2.2. The Experimental Design

The experiment was conducted during the 2022–2023 and 2023–2024 seasons. The experiment was conducted within a single commercial vineyard to ensure uniformity in soil composition, grape variety, vineyard management, and microclimatic conditions, thereby reducing confounding environmental variability. Each treatment comprised six consecutive vine rows separated by approximately 2–3 m from adjacent treatments to minimize potential cross-contamination among cover crops. To avoid edge effects and spatial autocorrelation, grape samples were collected exclusively from the four central rows of each treatment, while the two external rows were excluded. Grapes from these central rows were pooled to obtain a representative composite sample for each treatment, ensuring an integrated reflection of microbial variability within each block. From this homogenized pool, three independent small-scale fermentation replicates were prepared under identical winemaking conditions to ensure reproducibility. All fermentations were spontaneous, conducted without the use of commercial inocula or grapes from other treatments. This sampling strategy was designed to minimize intra-treatment variability and focus the analysis on differences attributable to the cover crop effect rather than micro-spatial or environmental gradients within the vineyard. Each treatment included 575 plants, covering approximately 0.138 hectares. Cover crops were sown in under-vine strips approximately 60–70 cm wide and consisted of the following management strategies (Treatments): (1) AG: agrochemical control, where glyphosate was applied under vines twice during the growing season (spring and summer) according to the manufacturer’s recommendations; (2) W: spontaneous vegetation (weeds such as *Convolvulus arvensis*, *Malva* sp., *Chenopodium albus*); (3) Lu: lupine (*Lupinus albus*) at a rate of 150 kg/ha, and (4) Bm: Burr medic (*Medicago polymorpha*) at a rate of 12 kg/ha, both crops sown in September 2022 and May 2023; (5) WC: white clover (*Trifolium repens*) at a rate of 8 kg/ha, and (6) RC: red clover (*Trifolium pratense*) at rate of 14 kg/ha, both established as a permanent cover crop under vines since September 2022.

### 2.3. Small-Scale Fermentation

*Cabernet Sauvignon* grapes were hand-harvested at 23° Brix and immediately transported to the winery. Grapes from sampled rows were combined to obtain three replicates per treatment, each consisting of approximately 20 kg of berries, yielding around 60 kg in total for microvinifications. Portions of 500 g of grapes were preserved at −20 °C for subsequent metagenomic analysis. After destemming and crushing, the must was placed in 50 L stainless steel tanks for fermentation. For each treatment, 40 L of wine were produced in triplicate. Spontaneous alcoholic fermentation was conducted at 26 ± 2 °C, with cap management through twice-daily punch-downs. No commercial yeast inoculation was performed; only acidity adjustment was applied prior to fermentation. When residual sugar levels stabilized below 4 g/L, the wines were racked off the skins, cold-stabilized for two weeks at 4 °C, and subsequently bottled.

### 2.4. Metagenomic Analysis

Microbial populations were evaluated for all experimental treatments in triplicate. 50 g of grapes were processed to analysis of bacteria and fungi. Aliquots of 50 mL were collected aseptically at three key fermentation stages initial (day 0), mid-fermentation (density 1045 g/mL), and final (density < 0.995 g/mL). A total of 54 microbial samples were immediately transferred into sterile sampling bags, transported to the laboratory under dry ice conditions, and stored at −80 °C until further analysis. DNA purification was carried out using the DNeasy PowerSoil Kit (Qiagen, Hilden, Germany), following the manufacturer’s protocol with slight modifications to improve inhibitor removal. DNA quantity and purity were assessed using a Nanodrop spectrophotometer and Qubit dsDNA HS assay (Thermo Fisher Scientific, Waltham, MA, USA). For bacterial analysis, the V4 region of the 16S rRNA gene was then amplified using primers 515F and 806R, as cited in reference [21]. For fungi analysis, primers fitS7/ITF4 were used as described [22]. The PCR mixture for each 30 μL reaction included 1.5 U of GoTaq^®^ G2 Flexi DNA Polymerase (Promega, Madison, WI, USA), 6 μL of buffer (5×), 1.5 mM of MgCl_2_, 0.25 pM of each primer, 0.5 mM dNTPs, and 20 ng of DNA. PCR runs also included negative controls without DNA. PCR products were purified using a QIAquick^®^ PCR Purification Kit (Qiagen, Hilden, Germany). Libraries were sequenced on the paired end Illumina platform Hiseq PE250 adapted for 300 bp paired-end reads at the CD Genomics. Amplicon sequence processing was performed in R (v4.4.0) using the DADA2 pipeline. Adapter and primer sequences were removed with Cutadapt. Reads were filtered and truncated based on quality scores, and amplicon sequence variants (ASVs) were inferred after error correction. Chimeras were removed, and taxonomy was assigned using the SILVA v138.1/UNITE v.10 databases with a naïve Bayesian classifier [23,24]. The resulting ASV table, taxonomy, and metadata were compiled into a phyloseq object for downstream analysis. Beta diversity was calculated using Aitchison distance and significant differences between the bacterial communities from different covers were evaluated using PERMANOVA. Additionally, the influence of dispersion among groups was determined through betadisper analysis (Permutation test for homogeneity of multivariate dispersions). The Aitchison distance was calculated as a metric of beta diversity. First, the transform function of the microbiome library [25] was used for the centered log transformation (clr) of the count table and then, using the vegan library [26], the Euclidean distance was calculated. The structure of the communities was represented by Principal Coordinate Analysis (PCoA). Spearman’s correlation analysis between abundance of genera and winemakers was evaluated using the Hmisc library [27].

### 2.5. General Enological Parameters

In the must, pH, titratable acidity (g L^−1^ tartaric acid), and soluble solids were measured in triplicate following standardized methods recommended by the International Organization of Vine and Wine [28]. Prior to fermentation, Yeast Assimilable Nitrogen (YAN) was also determined in triplicate, expressed as mg L^−1^ of nitrogen using OIV-approved protocols [28]. In the resulting wines, volatile acidity (g L^−1^ acetic acid), titratable acidity, alcohol content (% *v*/*v*), pH, color intensity, total polyphenol index, and total anthocyanins were analyzed in triplicate according to the same OIV standards [28], in order to evaluate their phenolic and chromatic characteristics [28].

### 2.6. Statistical Analyzes

Significant differences among treatments for each chemical parameter measured in must and wine were evaluated using a one-way analysis of variance (ANOVA) followed by Tukey’s post hoc test (*p* < 0.05) in GraphPad Prism software (version 10; GraphPad Software, San Diego, CA, USA). Prior to the ANOVA, data were tested for compliance with the assumptions of normality and homogeneity of variances. The Shapiro–Wilk test was applied to assess normality. As this assumption was met (*p* > 0.05), no data transformation was required.

## 3. Results

### 3.1. Fungal Community on Grapes

**Figure 2:**
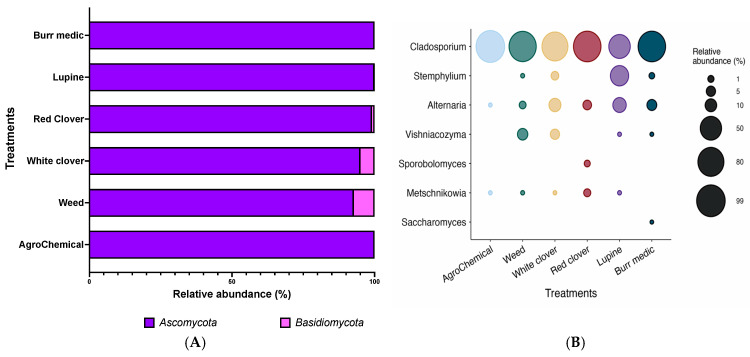
Composition (relative abundance) of fungi community on grapes. (**A**) Phylum-level composition in different treatments. (**B**) Genus-level composition in different treatments.

The fungal composition of grape samples from each treatment was mainly represented by two phyla: *Ascomycota* and *Basidiomycota* (Figure 2A). As shown in Table S1, *Ascomycota* was the dominant phylum, accounting for more than 90% of the relative abundance in all samples analyzed. In particular, the control, Bm, and Lu treatments exhibited relative abundances between 99.99% and 100%. Conversely, *Basidiomycota* was also widely distributed, although at lower levels (~5%). However, in the control, Bm, and Lu treatments, its relative abundance was minimal (0.01–0%).

At the genus level, the fungal composition of grape samples from each treatment was mainly represented by seven genera (Figure 2B; Table S2). Among these, *Cladosporium* was the most abundant genus in most samples, with values exceeding 80%. In the control, WC, and Bm treatments, its relative abundance reached over 90%, suggesting that *Cladosporium* is a dominant component of the grape-associated fungal microbiota. The genus *Metschnikowia* showed low relative abundance, ranging from 0.01% to 2% in the WC, Lu, W, and RC treatments (Table S2). In contrast, *Saccharomyces*, the most relevant yeast genus in fermentation processes, was not detected in most grape samples, exhibiting near-zero relative abundance across all treatments (Table S2). Similarly, the genera *Sporobolomyces* and *Stemphylium* were detected at very low levels, appearing only in the WC, Bm, and Lu treatments. In contrast, the genus *Vishniacozyma* displayed some variability, with relative abundances of 4.95% in WC and 7.25% in W, while in the remaining treatments and in the control its presence was practically negligible (Figure 2B).

### 3.2. Bacterial Community on Grapes

**Figure 3:**
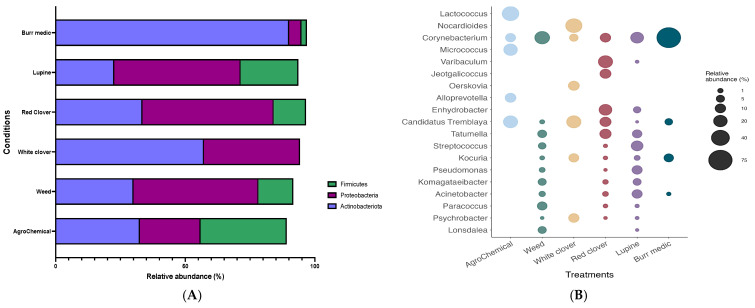
Composition (relative abundance) of bacterial community on grapes. (**A**) Phylum-level composition in different treatments. (**B**) Genus-level composition in different treatments.

The bacterial composition of grape samples from the different treatments was primarily represented by four phyla (Figure 3A; Table S3): Proteobacteria, Firmicutes, *Actinobacteriota*, and *Chloroflexi*. In general, the distribution of these phyla was similar across treatments, with a marked dominance of Proteobacteria. However, the Bm treatment exhibited a higher proportion of Actinobacteriota compared with the other treatments.

At the genus level, the bacterial composition of grape samples from each treatment comprised a total of 19 genera (Figure 3B; Table S4). The analysis of bacterial community composition revealed that the Lu, W, and Bm treatments shared the presence of the genus *Corynebacterium*, with similar relative abundances ranging between 15% and 25%. In contrast, the Bm treatment showed a substantially higher relative abundance of the same genus, reaching 77.26%. Conversely, the Lu, W, and RC treatments exhibited a higher abundance of the genus *Tatumella*, with values ranging from 6% to 12%. Finally, the control and WC treatments showed a predominant relative abundance of the genus *C. Tremblaya* (22%).

### 3.3. Dynamic of Fungal Community During Fermentation

**Figure 4:**
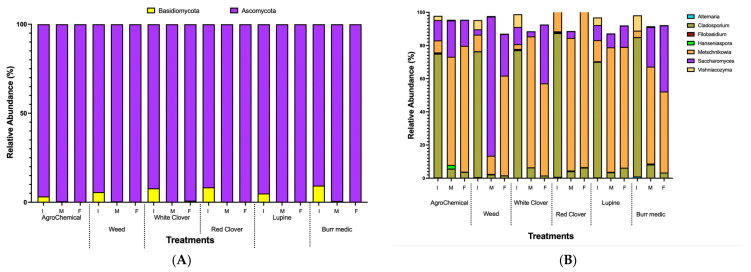
Relative abundance dynamics of dominant fungal during alcoholic fermentation (I. initial; M: medium; F: final) under different vineyard groundcover treatments. (**A**) phylum level; (**B**) genus level.

In this study, two fungal phyla were identified, with 90 and 10% of the annotated sequences classified as *Ascomycota* and *Basidiomycota*, respectively (Figure 4A, Table S5). Early fermentation stages were characterized by a more diverse microbial community, which progressively narrowed as *Ascomycota* yeasts became dominant.

The predominant fungal genera detected during alcoholic fermentation (AF) included *Alternaria*, *Cladosporium*, *Filobasidium*, *Hanseniaspora*, *Metschnikowia*, *Saccharomyces*, and *Vishniacozyma*. *Cladosporium* exhibited the highest relative abundance (75–85%) at the onset of AF across all treatments, but its proportion progressively decreased through the mid and final stages (Figure 4B, Table S6). Conversely, *Metschnikowia* displayed a consistent increase in relative abundance from mid-AF onwards. Notably, a decline in *Metschnikowia* was observed by the end of AF in the RC, Bm, and WC treatments, whereas in the W treatment its abundance continued to rise until the final stage, and in the control, it remained relatively stable between mid- and late AF. By the end of AF, non-*Saccharomyces* yeasts—particularly *Metschnikowia*—were more predominant than *Saccharomyces* across all treatments, underscoring the strong contribution of non-*Saccharomyces* species to the fermentative microbiota under these vineyard management conditions (Figure 4B).

### 3.4. Dynamic of Bacterial Community During Fermentation

**Figure 5:**
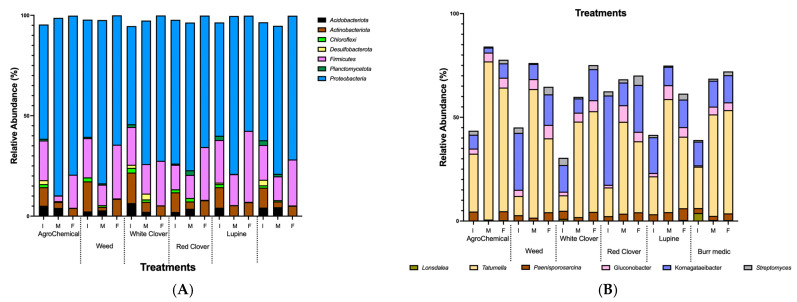
Relative abundance dynamics of dominant bacteria during alcoholic fermentation (I. initial; M: medium; F: final) under different vineyard groundcover treatments. (**A**) Phylum level; (**B**) genus level.

Seven bacterial phyla were identified *Proteobacteria*, *Firmicutes*, *Actinobacteriota*, *Acidobacteriota*, *Chloroflexi*, *Desulfobacterota*, and *Planctomycetota*. Figure 5A shows the relative abundance (%) of bacterial communities at the phylum level across treatments. The most predominant phyla were *Proteobacteria* (68.2%), *Firmicutes* (18.1%), and *Actinobacteriota* (7.16%), followed by lower proportions of *Acidobacteriota* (2.34%), *Desulfobacterota* (0.55%), *Chloroflexi* (0.87%), and *Planctomycetota* (0.68%). *Proteobacteria* dominated the community structure, whereas the contribution of the other phyla varied across samples (Table S7).

Among bacterial genera, *Tatumella* and *Komagataeibacter* were dominant across all stages of AF in most treatments (Figure 5B; Table S8). The relative abundance of *Tatumella* increased consistently from the beginning to the end of AF in WC and Bm treatments. Conversely, in Lu, W, RC and control, its abundance peaked at mid-AF and declined thereafter. *Komagataeibacter* showed a steady increase from the start to the end of AF in Bm and WC. In contrast, in W, Lu, RC treatments and control, its highest abundance was observed at mid-AF. Additionally, *Gluconobacter* displayed a notable increase in relative abundance from the beginning to the end of AF in the Bm, WC treatments and control, suggesting a continuous role in oxidative metabolism throughout fermentation.

### 3.5. Beta Diversity Analysis

**Figure 6:**
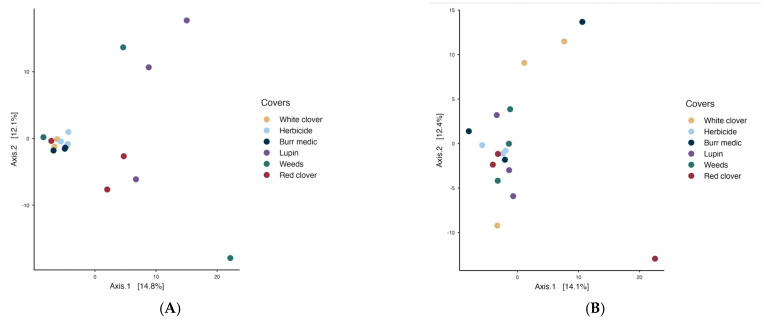
Beta diversity of grape-associated microbial communities: (**A**) bacterial assemblages and (**B**) yeast assemblages.

PERMANOVA analysis revealed no significant differences in microbial communities on the grape surface among treatments, indicating that bacterial (Figure 6) and yeast populations on grapes were similar (see Tables S9 and S10).

PERMANOVA analysis was also conducted to evaluate the temporal dynamics and differences in the structure of bacterial and yeast communities among vineyard ground cover treatments (WC, Control, Bm, Lu, W, and RC) across the course of spontaneous alcoholic fermentation. Community shifts were assessed at three fermentation stages: initial, intermediate, and final (see Tables S11 and S12).

**Figure 7:**
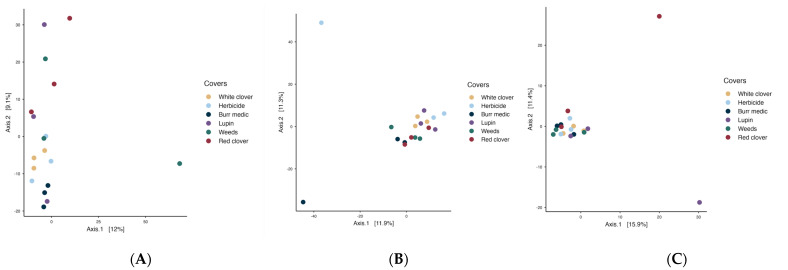
Beta-diversity of bacterial assemblages during the stages initial (**A**), intermediate (**B**) and final (**C**) of fermentation.

In the initial stage of spontaneous fermentation (Figure 7A), the global PERMANOVA value (*p* = 0.07) indicated no significant differences among treatments, suggesting a relatively homogeneous bacterial microbiota (Table S11). The betadisper value (*p* = 0.39) may indicate greater internal variability within the treatments. During the intermediate stage (Figure 7B), the overall PERMANOVA indicated a significant effect of treatment on bacterial community structure (*p* = 0.021), with no evidence of heterogeneous dispersion (betadisper *p* = 0.632). However, pairwise comparisons revealed no significant differences among treatments, suggesting that while some global compositional variation exists, bacterial communities remain largely similar across vineyard ground covers. In the final stage (Figure 7C), a similar result was observed (PERMANOVA *p* = 0.036; betadisper = 0.16).

**Figure 8:**
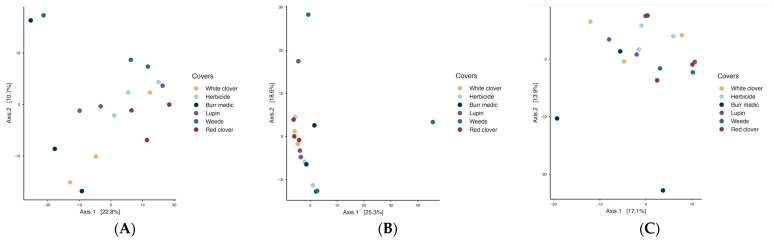
Beta-diversity of Fungi assemblages during the initial (**A**), intermediate (**B**) and final (**C**) stages of fermentation.

Regarding yeast assemblages PERMANOVA analysis revealed significant differences among covers during the initial stage (*p* = 0.018; betadisper = 0.539). Nevertheless, pairwise comparisons did not reveal significant differences among treatments, implying that despite minor global variation, the fungal (yeast) assemblages remained broadly consistent across vineyard ground cover types (Table S12). During the intermediate stage (Figure 8B), PERMANOVA detected significant differences in microbial community structure (*p* = 0.045), but the betadisper test (*p* = 0.042) indicated heterogeneous dispersion, implying that group variability may have influenced the observed separation. By the final stage (R^2^ = 0.316; *p* = 0.226; betadisper = 0.365), no significant differences in yeast composition were detected, and no internal variability among treatments was observed.

### 3.6. Must and Wine Physicochemical Parameters

**Figure 9:**
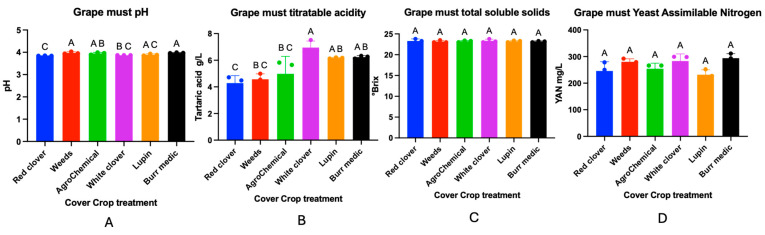
Must composition of *Cabernet Sauvignon* under different cover crop treatments. Panels represent the following analytical parameters: (**A**) Grape must pH, (**B**) Grape must titratable acidity, (**C**) Grape must total soluble solids, (**D**) Grape must Yeast assimilable Nitrogen (YAN). Statistical differences are represented by capital letter (A–C).

The effect of different cover crop treatments in a *Cabernet Sauvignon* vineyard on must composition was evaluated. The physicochemical parameters of the must are presented in Figure 9. No significant differences were observed in total soluble solids (TSS) or yeast assimilable nitrogen (YAN) among the treatments. In contrast, significant differences were detected in must pH values.

The highest pH values were recorded in the spontaneous weed (3.97 ± 0.02) and Burr medic (3.97 ± 0.01) treatments, which did not differ significantly from each other (group A). RC showed the lowest pH (3.84 ± 0), classified in group C, and differed significantly from the W and Bm treatments. AG (control) (3.94 ± 0.02), WC (3.87 ± 0), and Lu (3.88 ± 0.02) exhibited intermediate values, grouped into AB, BC, and AC, respectively, indicating no significant differences with some of the extreme treatments.

Significant differences in must titratable acidity were observed among treatments. The control, along with treatments W, WC, and A, formed a statistically homogeneous group (letter A), indicating a similar response in this parameter. Treatments Lu and Bm, classified as “AB”, showed intermediate values and did not differ significantly from either group A or treatment RC. The latter, assigned letter “C”, exhibited the most contrasting acidity values, differing significantly from group A. These findings suggest that certain treatments, particularly RC, may induce changes in must acidity, potentially influencing the acid balance of the final wine.

**Figure 10:**
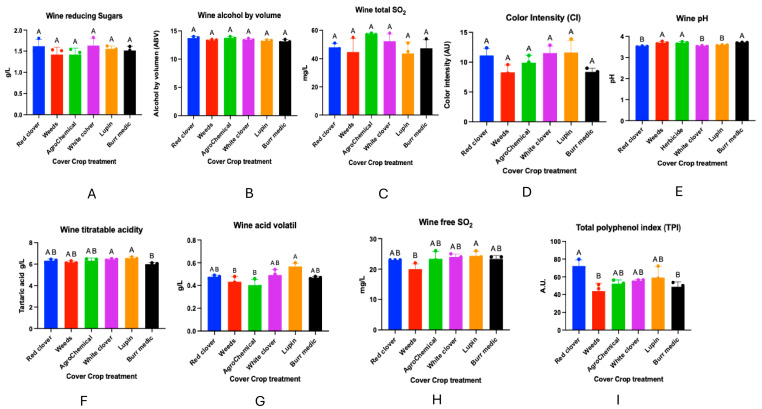
Wine composition of *Cabernet Sauvignon* under different cover crop treatments. Panels represent the following analytical parameters: (**A**) Wine reducing sugar, (**B**) Alcohol content, (**C**) Total SO_2_, (**D**) Color intensity, (**E**) pH, (**F**) Titratable acidity, (**G**) acid volatile, (**H**) Free SO, and (**I**) Total polyphenol index (TPI). These measurements summarize the main enological characteristics evaluated across treatments. Statistical differences are represented by capital letter (A, B).

Regarding the chemical analyses of the wines obtained from the different treatments, no significant differences were observed in reducing sugar content, alcoholic strength, total sulfur dioxide concentration, color intensity, or total anthocyanins (Figure 10). In contrast, significant differences were detected in wine pH. The highest values were recorded in treatments W (3.70 ± 0), Bm (3.70 ± 0.00), and control (3.69 ± 0.03), which did not differ significantly from each other (group A). On the other hand, RC (3.52 ± 0.01), WC (3.54 ± 0.01), and Lu (3.58 ± 0.01) were classified in group B.

Significant differences were also found in total acidity. Treatments WC (6.45 ± 0.04) and Lu (6.54 ± 0.07) showed the highest values, forming group A. Intermediate values were observed for RC (6.32 ± 0.09), W (6.18 ± 0.06), and control (6.32 ± 0.15), grouped into AB. The lowest value was obtained in Bm (6.00 ± 0.08), classified in group B.

With respect to volatile acidity, significant differences were observed among treatments. RC (0.47 ± 0.02), WC (0.49 ± 0.02), Lu (0.56 ± 0.01), and Bm (0.47 ± 0.01) exhibited intermediate values (group AB), showing no significant differences with some of the extreme treatments. In contrast, W (0.43 ± 0.01) and control (0.40 ± 0.29) presented the lowest values, grouped into B.

Regarding free SO_2_ concentration, treatment W showed the lowest value (22.00 ± 1.00), classified into group B, and differed significantly from RC (23.00 ± 0), control (23.33 ± 1.45), WC (24.00 ± 0.57), Lu (24.33 ± 0.88), and Bm (23.33 ± 0.66), which exhibited intermediate values (group AB).

Finally, the total polyphenol index showed significant differences among treatments. RC presented the highest concentration (72.3 ± 3.92), forming group A. Intermediate values were recorded for WC (55.8 ± 0.8), control (52.23 ± 2.58), and Lu (59.18 ± 7.31), grouped into AB. The lowest value was observed in Bm (48.83 ± 3.23), corresponding to group B.

### 3.7. Correlation Between Microbial Population and Wine Parameters

**Figure 11:**
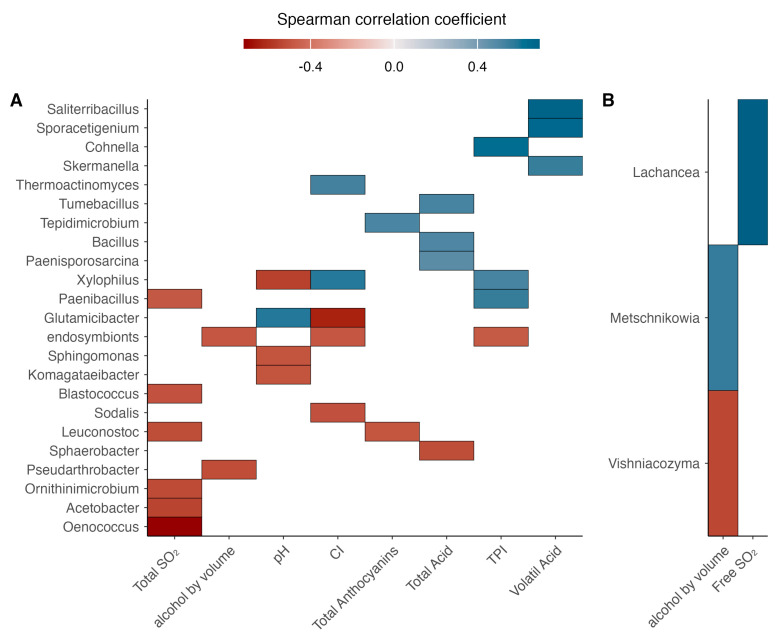
Heatmap of Spearman’s correlation coefficients (ρ) between microbial populations and the physicochemical parameters of wine. (**A**) correspond to bacterial taxa correlations; (**B**) correspond to fungi taxa correlations. The color scale represents correlation strength, ranging from −1 (red, strong negative correlation) to +1 (blue, strong positive correlation). Significant correlations (*p* < 0.05) are highlighted.

The correlation analysis between the relative abundance of microbial populations and wine chemical parameters was represented using a Spearman correlation heatmap (Figure 11), showing both positive and negative associations of high and moderate strength. The strongest positive correlations involved *Saliterribacillus*, and *Sporacetigenium* with volatile acidity, while *Cohnella* was positively associated with the total polyphenol index (r = 0.4). Regarding negative correlations, the strongest associations included *Oenococcus* with free SO_2_, *Glutamicibacter* with color intensity, and *Xylophilus* with pH.

For yeast populations, the most notable positive correlations were observed for *Lachancea* with free SO_2_, while *Metschnikowia* showed a moderate positive correlation with ethanol content. Conversely, *Vishniacozyma* exhibited a moderate negative correlation with ethanol concentration.

## 4. Discussion

The results of this study demonstrate that leguminous cover crops exert selective effects on both the chemical composition and microbial dynamics of *Cabernet Sauvignon* must and wine. In the must, pH values were higher in treatments with white clover (WC), Burr medic (Bm), and spontaneous weeds (W), whereas titratable acidity (TA) was significantly elevated under red clover (RC), indicating that cover crops influence grape acid metabolism. No significant differences were observed in total soluble solids (TSS) or yeast-assimilable nitrogen (YAN), consistent with previous reports showing that effective nitrogen transfer from leguminous cover crops to grapes is highly variable and depends on species, nitrogen mineralization dynamics, and water competition [29,30,31]. Previous studies involving *Trifolium pratense*, *Lupinus* spp., and *Medicago polymorpha* likewise reported stable total soluble solids (TSS) levels in *Cabernet Sauvignon*, consistent with our observations under Mediterranean conditions in Chile [32,33,34].

In wines, significant differences were found in pH, TA, volatile acidity, free SO_2_, and total polyphenol index. The highest total polyphenol index was observed in red clover (RC) treatments, which may be attributed to both concentration effects and moderate stress-induced stimulation of phenolic compound synthesis. Although few studies have focused specifically on red clover, existing research on clover cover crops indicates that they can reduce vegetative growth, enhance cluster light exposure, and promote phenolic compound accumulation, thereby positively influencing wine quality [30,33]. Legume-based treatments also exhibited lower wine pH and higher TA, whereas Burr medic and spontaneous weed treatments tended to produce higher pH values, likely reflecting improvements in soil fertility, nitrogen cycling, and microbial interactions associated with legume-based systems. No significant effects were detected for reducing sugars, alcohol content, total SO_2_, color intensity, or total anthocyanins, underscoring that the impact of cover crops can vary considerably depending on the plant species used, local climatic conditions, and vineyard management strategies [35].

Microbiologically, vineyard groundcover management did not induce significant differences in the overall structure of bacterial or fungal communities on grape berries, as indicated by PERMANOVA analyses. This finding supports previous reports suggesting that the fruit microbiome is more strongly shaped by microclimatic factors, biological vectors, and canopy conditions than by soil management alone [6,7]. Nevertheless, the relative abundance of specific genera, such as *Corynebacterium* in Burr medic and *Tatumella* in lupine and weed treatments, indicates that inter-row covers can exert subtle yet consistent selective pressures on certain microbial groups. Although PERMANOVA indicated no statistically significant differences among treatments, the partial separation of Burr medic and Lupine samples in the PCoA plots (Figure 6 and Figure 7) may reflect fine-scale environmental or biochemical influences of these cover crops. Differences in root exudate composition, nitrogen fixation capacity, and canopy microclimate could modulate the abundance of specific bacterial groups, as observed for leguminous covers in Mediterranean vineyards [4,11,29,32,33]. Such micro-scale variations, while insufficient to generate distinct community clusters, may nonetheless shape transient microbial assemblages on grape surfaces during ripening. These observations align with studies showing that cover crops may modify microbial composition by influencing humidity, organic matter input, and local temperature gradients [4,11]. Thus, rather than disrupting the vineyard microbiome, leguminous covers appear to modulate its taxonomic and functional balance while maintaining overall ecosystem stability.

During fermentation, the fungal community showed a predictable successional dynamic characterized by the dominance of *Ascomycota*, particularly the non-*Saccharomyces* genera *Metschnikowia* and *Hanseniaspora*, followed by *Saccharomyces* at later stages [1,2,4]. These results reflect the natural progression of spontaneous fermentations, where initial diversity narrows as ethanol concentrations rise [1,2,4]. Interestingly, *Metschnikowia* remained dominant in several treatments through the final fermentation stages, suggesting that the epiphytic microbiota shaped by sustainable groundcovers may contribute to a longer persistence of non-*Saccharomyces* yeasts. Such persistence can influence fermentation kinetics and secondary metabolite formation, supporting the idea that vineyard management indirectly affects wine sensory outcomes by modulating the native microbial terroir [36]. Similar findings have been reported in Mediterranean vineyards managed under ecological or cover crop systems [11], which promote stable microbial succession and functional redundancy during fermentation.

Although *Metschnikowia* species are typically regarded as low-ethanol-tolerant yeasts, generally losing viability above 4–6% (*v*/*v*) ethanol [37], certain isolates have demonstrated moderate resistance up to 8–9% (*v*/*v*) [37,38,39] and even 11% (*v*/*v*) under specific fermentation conditions or in selected commercial strains [39]. Therefore, the observed positive correlation between *Metschnikowia* abundance and ethanol levels in this study may indicate either the presence of a particularly tolerant strain or ecological interactions that enhance its persistence during spontaneous fermentation, such as co-metabolism with *Saccharomyces* or protective matrix effects within the microbial consortium. Similarly, Ref. [40] indicated that *M. pulcherrima* contributed to the wine’s aromatic profile during fermentation and aging, suggesting its persistence and metabolic activity in the final stages of the process.

The correlation analysis provided new insight into potential functional linkages between microbial abundance and wine physicochemical attributes. Positive associations between *Saliterribacillus* and volatile acidity suggest that certain bacterial taxa may coexist with or respond to conditions that favor minor acidification processes or acetoin metabolism during fermentation. Members of the genus *Saliterribacillus* belong to the family *Bacillaceae* and are moderately halophilic, spore-forming bacteria capable of producing organic acids and volatile metabolites through carbohydrate fermentation [41]. Their metabolic flexibility and tolerance to osmotic stress could enable survival in the high-sugar, saline environments of grape must, where they might contribute indirectly to redox processes that influence the volatile balance of wine. Similarly, *Cohnella* showed a moderate positive correlation with the total polyphenol index, which could reflect either a shared response to phenolic-rich environments or a potential role in the transformation of phenolic compounds. Members of *Cohnella* are aerobic or facultatively anaerobic spore-forming bacteria within the *Bacillaceae* family, known for producing extracellular enzymes such as cellulases, β-glucosidases, and polyphenol oxidases [42,43]. These enzymes are capable of hydrolyzing plant polysaccharides and phenolic glycosides, processes that could, in theory, facilitate the release of bound phenolic compounds during maceration and fermentation. Indeed, certain *Cohnella* strains exhibit phenol-transforming activity linked to their enzymatic repertoire [44]. Although these correlations do not demonstrate causality, the recurrent detection of *Cohnella* in grape and wine environments [2,4] is consistent with the hypothesis that members of this genus may influence, directly or indirectly, the phenolic dynamics and color stability of wine through enzymatic or ecological interactions.

Among yeasts, the positive correlation of *Metschnikowia* with ethanol content highlights its fermentative contribution under spontaneous conditions, while *Vishniacozyma* showed an inverse trend, consistent with its oxidative rather than fermentative metabolism. *Metschnikowia* species are recognized for their high metabolic versatility, secreting enzymes such as β-glucosidases, esterases, and proteases that enhance the release of bound aroma precursors and contribute to the formation of volatile esters and higher alcohols during fermentation [39]. Beyond these fermentative functions, the persistence of *Metschnikowia pulcherrima* observed during the mid and late stages of spontaneous alcoholic fermentation may be explained by complementary physiological and ecological mechanisms. This yeast synthesizes pulcherrimin and pulcherriminic acid—iron-chelating compounds that reduce metal availability to other microorganisms and modulate community composition [45,46]. These metabolites, together with its ability to form biofilms and produce extracellular enzymes (β-glucosidases, pectinases, lipases), enhance stress tolerance and bioprotective capacity, creating a metabolically favorable microenvironment [47,48]. Such traits could explain its maintenance beyond the early fermentation phases, as previously observed under restrictive oenological conditions [48,49]. Altogether, these features portray *M. pulcherrima* as a metabolically active and ecologically resilient species that contributes to aroma development, community stability, and the overall biochemical balance of spontaneous fermentations. Conversely, *Vishniacozyma* (formerly *Cryptococcus*) is primarily oxidative, contributing to redox balance through catalase and polyphenol oxidase activities that may influence wine color stability and phenolic oxidation [50]. These functional contrasts underscore how yeast community structure mirrors ongoing biochemical processes during fermentation, reinforcing the potential of microbial profiles as biomarkers of enological quality and vineyard microbial diversity.

Physicochemical analyses revealed that cover crop treatments did not significantly alter major enological parameters such as sugar concentration or alcohol level, yet they influenced must and wine pH, acidity, and total polyphenol index. In particular, treatments using leguminous covers—such as white and red clover or lupine—exhibited lower wine pH and higher total acidity compared to the herbicide-treated control, while Burr medic and spontaneous weed treatments tended to produce higher pH values. These differences may reflect improved soil fertility, nitrogen cycling, and microbial interactions associated with legume-based systems, which can enhance nutrient availability and acid balance in grapes. The higher polyphenol content observed in red clover treatments further supports the hypothesis that sustainable ground management not only preserves microbial diversity but also contributes to the phenolic and sensory complexity of the resulting wines. Overall, these findings demonstrate that groundcovers can promote enological quality without compromising fermentation performance, while reducing reliance on chemical herbicides.

## 5. Conclusions

Collectively, these findings indicate that leguminous cover crops selectively modulate chemical and microbial attributes of must and wine, promoting enological quality without compromising fermentation performance. The vineyard microbiome exhibits resilience to cover crop management, maintaining a stable “microbial terroir” while allowing targeted adjustments that contribute to wine sensory complexity. From an ecological perspective, cover crops enhance biodiversity and soil regeneration, reinforcing the link between vineyard ecosystem and wine identity. Future studies integrating metabolomic and transcriptomic analyses will be essential to identify microbial functions and metabolites responsible for the observed enological differentiation, supporting cover crops as a sustainable viticultural strategy in Mediterranean climates such as the Maipo Valley.

## Data Availability

The raw data supporting the conclusions of this article will be made available by the authors on request.

## Supplementary Materials

The following supporting information can be downloaded at: https://www.mdpi.com/article/10.3390/microorganisms13122804/s1, Supp Mat Beta diversity.docx—Contains PERMANOVA and Betadisper results for bacterial (16S) and fungal (ITS) communities. Tables S9 and S10 summarize grape sample comparisons across vineyard treatments, and Tables S11 and S12 show equivalent analyses for microfermentation stages (Initial, Medium, Final). Supp Mat Abundance.xls—Contains relative abundance data for bacterial (16S) and fungal (ITS) communities. Tables S1–S8 summarize the mean relative abundance of dominant phyla and genera across vineyard treatments and fermentation stages, providing quantitative support for the community structure analyses presented in the main text.

## Abbreviations

The following abbreviations are used in this manuscript:

16S rRNA	16S ribosomal ribonucleic acid
AF	Alcoholic fermentation
AG	Agrochemical control
ANOVA	One-way analysis of variance
ASVs	Amplicon Sequence Variants
Bm	Burr medic (*Medicago polymorpha*)
DADA2 pipeline	Denoising Algorithm for Amplicon Data version 2 pipeline
DNA	Deoxyribonucleic acid
dNTPs	Deoxynucleotide triphosphates
ITS	Internal Transcribed Spacer
Lu	Lupine (*Lupinus albus*)
mid-AF	Middle alcoholic fermentation
OIV	Organization of Vine and Wine
PCR	Polymerase Chain Reaction
PERMANOVA	Permutational Multivariate Analysis of Variance
PCoA	Principal Coordinate Analysis
RC	Red clover (*Trifolium pratense*)
SO_2_	Sulfur dioxide
TSS	Total Soluble Solids
*v*/*v*	Volume per volume
W	Spontaneous vegetation (weeds such as *Convolvulus arvensis*, *Malva* sp., *Chenopodium albus*)
WC	White clover (*Trifolium repens*)
YAN	Yeast Assimilable Nitrogen

## References

[1] Barata A., Malfeito-Ferreira M., Loureiro V. (2012). The Microbial Ecology of Wine Grape Berries. Int. J. Food Microbiol..

[2] Morrison-Whittle P., Goddard M.R. (2018). From Vineyard to Winery: A Source Map of Microbial Diversity Driving Wine Fermentation. Environ. Microbiol..

[3] Zarraonaindia I., Owens S.M., Weisenhorn P., West K., Hampton-Marcell J., Lax S., Bokulich N.A., Mills D.A., Martin G., Taghavi S. (2015). The Soil Microbiome Influences Grapevine-Associated Microbiota. mBio.

[4] Belda I., Zarraonaindia I., Perisin M., Palacios A., Acedo A. (2017). From Vineyard Soil to Wine Fermentation: Microbiome Approximations to Explain the “Terroir” Concept. Front. Microbiol..

[5] Comitini F., Ciani M. (2008). Influence of Fungicide Treatments on the Occurrence of Yeast Flora Associated with Wine Grapes. Ann. Microbiol..

[6] Chou M.Y., Vanden Heuvel J., Bell T.H., Panke-Buisse K., Kao-Kniffin J. (2018). Vineyard Under-Vine Floor Management Alters Soil Microbial Composition, While the Fruit Microbiome Shows No Corresponding Shifts. Sci. Rep..

[7] Mandl K., Cantelmo C., Gruber E., Faber F., Friedrich B., Zaller J.G. (2018). Effects of Glyphosate-, Glufosinate-, and Flazasulfuron-Based Herbicides on Soil Microorganisms in a Vineyard. Bull. Environ. Contam. Toxicol..

[8] Wu H., Su X., Singh V.P., Feng K., Niu J. (2021). Agricultural Drought Prediction Based on Conditional Distributions of Vine Copulas. Water Resour. Res..

[9] Agarbati A., Canonico L., Ciani M., Comitini F. (2019). The Impact of Fungicide Treatments on Yeast Biota of Verdicchio and Montepulciano Grape Varieties. PLoS ONE.

[10] Chen N., Wei R., Cao X., Duan X., Li H., Wang H. (2022). Evaluation of Inter-Row Cover Crops Effects on the Microbial Diversity during Cabernet Sauvignon (*Vitis vinifera* L.) Maturation. Food Res. Int..

[11] Rocha F.I., Rodriguez-Ramos J.C., Fernando M., Hale L. (2025). Interrow Cover Crops in a Semi-Arid Vineyard Increase Plant-Beneficial Functional Potential of the Soil Microbiome, Both in Vine Rows and Interrows, a Benefit That Increases with Cover Crop Duration. Environ. Microbiome.

[12] Wang Y., Gao X.T., Li H.Q., Lu H.C., He L., Peng W.T., Wang J. (2021). Microclimate Changes Caused by Black Inter-Row Mulch Decrease Flavonoid Concentrations in Grapes and Wines under Semi-Arid Climate. Food Chem..

[13] Bokulich N.A., Thorngate J.H., Richardson P.M., Mills D.A. (2014). Microbial Biogeography of Wine Grapes Is Conditioned by Cultivar, Vintage, and Climate. Proc. Natl. Acad. Sci. USA.

[14] Mezzasalma V., Sandionigi A., Guzzetti L., Galimberti A., Grando M.S., Tardaguila J., Labra M. (2018). Geographical and Cultivar Features Differentiate Grape Microbiota in Northern Italy and Spain Vineyards. Front. Microbiol..

[15] He F., Tian M.-B., Duan W.-P., Yang W.-M., Mao X., Wang J., Duan C.-Q. (2023). Effects of Inner-Row Ground Management on the Volatomics of ‘Cabernet Sauvignon’ Grapes and Wines in the Region of the Eastern Foothills of the Ningxia Helan Mountains in Northwest China. Foods.

[16] Sáez V., Schober D., González Á., Arapitsas P. (2021). LC–MS-Based Metabolomics Discriminates Premium from Standard Chilean cv. Cabernet Sauvignon Wines from Different Valleys. Metabolites.

[17] Charlin V., Cifuentes A. (2025). The Effects of Climate Change on the Quality of Chile’s Maipo Valley Cabernet Sauvignon Wines. J. Wine Res..

[18] Marques M.J., García-Muñoz S., Muñoz-Organero G., Bienes R. (2010). Soil Conservation beneath Grass Cover in Hillside Vineyards under Mediterranean Climatic Conditions (Madrid, Spain). Land Degrad. Dev..

[19] Novara A., Catania V., Tolone M., Gristina L., Laudicina V.A., Quatrini P. (2020). Cover Crop Impact on Soil Organic Carbon, Nitrogen Dynamics and Microbial Diversity in a Mediterranean Semiarid Vineyard. Sustainability.

[20] Celette F., Findeling A., Gary C. (2009). Competition for Nitrogen in an Unfertilized Intercropping System: The Case of an Association of Grapevine and Grass Cover in a Mediterranean Climate. Eur. J. Agron..

[21] Romero J., Catalán N., Ramírez C., Miranda C.D., Oliva M., Flores H., Romero M.S., Rojas R. (2023). High Abundance of *Candidatus* Arthromitus in Intestinal Microbiota of *Seriolella violacea* (Palm Ruff) under Reared Conditions. Fishes.

[22] Ihrmark K., Bödeker I.T., Cruz-Martinez K., Friberg H., Kubartova A., Schenck J., Strid Y., Stenlid J., Brandström-Durling M., Clemmensen K.E. (2012). New Primers to Amplify the Fungal ITS2 Region—Evaluation by 454-Sequencing of Artificial and Natural Communities. FEMS Microbiol. Ecol..

[23] Quast C., Pruesse E., Yilmaz P., Gerken J., Schweer T., Yarza P., Peplies J., Glöckner F.O. (2013). The SILVA Ribosomal RNA Gene Database Project: Improved Data Processing and Web-Based Tools. Nucleic Acids Res..

[24] Abarenkov K., Zirk A., Piirmann T., Pöhönen R., Ivanov F., Nilsson R.H., Kõljalg U. (2025). UNITE General FASTA Release for Eukaryotes 2; UNITE Community. https://unite.ut.ee/repository.php.

[25] Lahti L., Shetty S. (2017). Microbiome: R Package; Bioconductor. https://bioconductor.org/packages/release/bioc/html/microbiome.html.

[26] Oksanen J., Simpson G.L., Blanchet F.G., Kindt R., Legendre P., Minchin P.R., O’Hara R.B., Solymos P., Stevens M.H.H., Szoecs E. *Vegan: Community Ecology Package*, R Package Version 2017. https://cran.r-project.org/web/packages/vegan/index.html.

[27] Harrell F.E. *Hmisc: Harrell Miscellaneous*, R Package Version 5.2-1; 2024. https://cran.r-project.org/package=Hmisc.

[28] International Organisation of Vine and Wine (OIV) (2012). Compendium of International Methods of Wine and Must Analysis.

[29] Ovalle C., del Pozo A. (2010). Estimating the Contribution of Nitrogen from Legume Cover Crops to the Nitrogen Nutrition of Grapevines Using a ^15^N Dilution Technique. Plant Soil.

[30] Giese G., Velasco-Cruz C., Roberts L., Heitman J., Wolf T.K. (2014). Complete Vineyard Floor Cover Crops Favorably Limit Grapevine Vegetative Growth. Sci. Hortic..

[31] Pisciotta A., Di Lorenzo R., Novara A., Laudicina V.A., Barone E., Santoro A., Gristina L., Barbagallo M.G. (2021). Cover Crop and Pruning Residue Management to Reduce Nitrogen Mineral Fertilization in Mediterranean Vineyards. Agronomy.

[32] Sulas L., Mercenaro L., Campesi G., Nieddu G. (2017). Different Cover Crops Affect Nitrogen Fluxes in Mediterranean Vineyard. Agron. J..

[33] Abad J., de Mendoza I.H., Marín D., Orcaray L., Santesteban L.G. (2021). Cover Crops in Viticulture. A Systematic Review (2): Implications on Vineyard Agronomic Performance. OENO One.

[34] Bettoni J.C., Stefanello L.O., Fogaça A.O. (2016). Influence of Different Soil Management Systems on Grapevine Performance in Southern Brazil. Rev. Bras. Frutic..

[35] Peng J., Wei W., Lu H.-C., Chen W., Li S.-D., Wang J., Duan C.-Q., He F. (2022). Effect of Covering Crops between Rows on the Vineyard Microclimate, Berry Composition, and Wine Sensory Attributes of ‘Cabernet Sauvignon’ (*Vitis vinifera* L. cv.) Grapes in a Semi-Arid Climate of Northwest China. Horticulturae.

[36] Jara C., Laurie V.F., Mas A., Romero J. (2016). Microbial Terroir in Chilean Valleys: Diversity of Non-Conventional Yeast. Front. Microbiol..

[37] Vicente J., Ruiz J., Belda I., Benito-Vázquez I., Marquina D., Calderón F., Santos A., Benito S. (2020). The Genus *Metschnikowia* in Enology. Microorganisms.

[38] Pawlikowska E., James S.A., Breierova E., Antolak H., Kregiel D. (2019). Biocontrol Capability of Local *Metschnikowia* sp. Isolates. Antonie Van Leeuwenhoek.

[39] Canonico L., Comitini F., Ciani M. (2019). *Metschnikowia pulcherrima* Selected Strain for Ethanol Reduction in Wine: Influence of Cell Immobilization and Aeration Condition. Foods.

[40] Torres-Díaz L.L., Murillo-Peña R., Iribarren M., Sáenz de Urturi I., Marín-San Román S., González-Lázaro M., Pérez-Álvarez E.P., Garde-Cerdán T. (2024). Exploring *Metschnikowia pulcherrima* as a Co-Fermenter with *Saccharomyces cerevisiae*: Influence on Wine Aroma during Fermentation and Ageing. Beverages.

[41] Amoozegar M.A., Bagheri M., Didari M., Fazeli S.A.S., Schumann P., Sánchez-Porro C., Ventosa A. (2013). *Saliterribacillus persicus* gen. nov., sp. nov., a Moderately Halophilic Bacterium Isolated from a Hypersaline Lake. Int. J. Syst. Evol. Microbiol..

[42] Kim J., Chhetri G., Kim I., Kang M., Seo T. (2021). *Cohnella terricola* sp. nov., Isolated from Soil. Int. J. Syst. Evol. Microbiol..

[43] Harirchi S., Sar T., Ramezani M., Aliyu H., Etemadifar Z., Nojoumi S.A., Yazdian F., Awasthi M.K., Taherzadeh M.J. (2022). *Bacillales*: From Taxonomy to Biotechnological and Industrial Perspectives. Microorganisms.

[44] Morya R., Kumar M., Thakur I.S. (2019). *Cohnella* sp. A01: A Novel Bacterium for the Efficient Production of Laccase for Application in Decolorization and Detoxification of Industrial Dyes. Heliyon.

[45] Sipiczki M. (2020). *Metschnikowia pulcherrima* and Related Pulcherrimin-Producing Yeasts: Fuzzy Species Boundaries and Complex Antimicrobial Antagonism. Microorganisms.

[46] Puyo M., Agnolucci M., Comitini F., Ciani M. (2023). Bio-Protection in Oenology by *Metschnikowia pulcherrima*: Mechanisms, Benefits and Challenges. Front. Microbiol..

[47] Charron-Lamoureux V., Duchesne I., Boisvert S., Barbeau X., Turcotte F., Labrie S. (2023). Pulcherriminic Acid Modulates Iron Availability and Protects Yeasts from Oxidative Stress through Biofilm Formation. Nat. Commun..

[48] Agarbati A., Canonico L., Ciani M., Comitini F. (2023). *Metschnikowia pulcherrima* in Cold Clarification: Biocontrol Potential and Enzymatic Contribution to Volatile Precursors. Fermentation.

[49] Testa B., Campolongo S., Russo P., Succi M., Tremonte P. (2024). Preliminary Characterization of *Metschnikowia pulcherrima* Strains for Oenological Application as Starter Cultures. Beverages.

[50] Maicas S., Mateo J.J. (2023). The Life of Saccharomyces and Non-Saccharomyces Yeasts in Drinking Wine. Microorganisms.

