# Supplementary material for: From Soil to Wine: Influence of Vegetative Covers on Microbial Communities and Fermentative Dynamics in Cabernet Sauvignon
Source: Microorganisms. 2025 Dec 9;13(12):2804. doi: 10.3390/microorganisms13122804 (PMC12735590; doi:10.3390/microorganisms13122804)
Supplement: Supplementary file 1 [file microorganisms-13-02804-s001.zip › Supp Mat Beta diversity.pdf]

BETA DIVERSITY GRAPES

Aitchison

Table S9. Comparison bacteria communities

	PERMANOVA		Betadisper
	R^2	*P* value	*P* value
All	0.324	**0.033**	**0.001**
Pairwise comparison			
Control vs Agrochemical	0.179	0.8	0.458
Control vs White clover	0.197	0.3	0.833
Control vs Lupine	0.275	0.1	**0.009**
Control vs Weed	0.218	0.1	**0.004**
Control vs Red clover	0.208	0.5	**0.036**
Agrochemical vs White clover	0.233	0.4	0.844
Agrochemical vs Lupine	0.272	0.1	**0.018**
Agrochemical vs Weed	0.214	0.2	**0.01**
Agrochemical vs Red clover	0.206	0.4	**0.028**
White clover vs Lupine	0.274	0.1	0.074
White clover vs Weed	0.231	0.1	0.05
White clover vs Red clover	0.230	0.2	0.09
Lupine vs Weed	0.191	0.7	0.255
Lupine vs Red clover	0.220	0.2	0.413
Weed vs Red clover	0.190	0.8	0.116

Tabla S10. Comparison Fungi communities

	PERMANOVA		Betadisper
	R^2	*P* value	*P* value
All	0.290	0.569	0.497
Pairwise comparison			
Control vs Agrochemical	0.216	0.4	0.201
Control vs White clover	0.165	0.9	0.816
Control vs Lupine	0.189	0.6	0.137
Control vs Weed	0.173	0.8	0.12
Control vs Red clover	0.193	0.6	0.907
Agrochemical vs White clover	0.196	0.8	0.48
Agrochemical vs Lupine	0.230	0.1	0.803
Agrochemical vs Weed	0.194	1.0	0.978
Agrochemical vs Red clover	0.195	0.7	0.291
White clover vs Lupine	0.225	0.1	0.379
White clover vs Weed	0.179	1.0	0.428
White clover vs Red	0.187	0.7	0.776

clover			
Lupine vs Weed	0.236	0.2	0.797
Lupine vs Red clover	0.210	0.4	0.233
Weed vs Red clover	0.192	0.8	0.263

BETA DIVERSITY FERMENTATION

Aitchison

Table S11. Summary beta diversity bacteria

	PERMANOVA		Betadisper
**Initial**	R^2	*P* value	*P* value
All	0.313	0.07	0.39
Pairwise comparison			
Control vs Agrochemical	0.178	0.7	0.09
Control vs White clover	0.233	0.1	**0.003**
Control vs Lupine	0.208	0.3	0.47
Control vs Weed	0.218	0.1	0.617
Control vs Red clover	0.223	0.1	0.775
Agrochemical vs White clover	0.247	0.1	0.757
Agrochemical vs Lupine	0.205	0.3	0.241
Agrochemical vs Weed	0.215	0.1	0.265
Agrochemical vs Red clover	0.227	0.1	0.594
White clover vs Lupine	0.227	0.2	**0.017**
White clover vs Weed	0.217	0.1	0.189
White clover vs Red clover	0.260	0.1	0.404
Lupine vs Weed	0.194	0.7	0.487
Lupine vs Red clover	0.183	0.8	0.913
Weed vs Red clover	0.190	0.7	0.518
**medium**			
All	0.316	**0.021**	0.632
Pairwise comparison			
Control vs Agrochemical	0.209	0.4	0.404
Control vs White clover	0.233	0.1	0.061
Control vs Lupine	0.194	0.6	0.874
Control vs Weed	0.206	0.4	**0.017**
Control vs Red clover	0.219	0.1	0.063
Agrochemical vs White clover	0.234	0.2	0.98
Agrochemical vs Lupine	0.211	0.3	0.511
Agrochemical vs Weed	0.216	0.3	0.792
Agrochemical vs Red clover	0.230	0.1	0.874
White clover vs Lupine	0.245	0.1	0.29

White clover vs Weed	0.199	0.6	0.54
White clover vs Red clover	0.211	0.3	0.777
Lupine vs Weed	0.221	0.1	0.409
Lupine vs Red clover	0.230	0.1	0.35
Weed vs Red clover	0.185	1	0.708
**Final**			
All	0.321	**0.036**	0.16
Pairwise comparison			
Control vs Agrochemical	0.207	0.3	0.336
Control vs White clover	0.246	0.1	0.165
Control vs Lupine	0.235	0.1	0.043
Control vs Weed	0.227	0.3	0.361
Control vs Red clover	0.192	0.6	0.159
Agrochemical vs White clover	0.220	0.3	0.661
Agrochemical vs Lupine	0.235	0.1	0.081
Agrochemical vs Weed	0.247	0.2	0.916
Agrochemical vs Red clover	0.217	0.2	0.255
White clover vs Lupine	0.230	0.1	0.151
White clover vs Weed	0.186	0.6	0.807
White clover vs Red clover	0.220	0.3	0.399
Lupine vs Weed	0.225	0.2	0.105
Lupine vs Red clover	0.210	0.4	0.767
Weed vs Red clover	0.212	0.4	0.315

Table S12. Summary beta diversity Fungi

	PERMANOVA		Betadisper
**Initial**	R^2	*P* value	*P* value
All	0.364	**0.018**	0.539
Pairwise comparison			
Control vs Agrochemical	0.214	0.4	0.532
Control vs White clover	0.223	0.2	0.27
Control vs Lupine	0.178	0.5	0.879
Control vs Weed	0.258	0.2	0.969
Control vs Red clover	0.257	0.3	0.247
Agrochemical vs White clover	0.308	0.1	0.095
Agrochemical vs Lupine	0.218	0.4	0.449
Agrochemical vs Weed	0.252	0.2	0.732
Agrochemical vs Red	0.289	0.1	0.569

clover			
White clover vs Lupine	0.249	0.1	0.055
White clover vs Weed	0.260	0.3	0.519
White clover vs Red clover	0.356	0.1	**0.019**
Lupine vs Weed	0.202	0.4	0.987
Lupine vs Red clover	0.238	0.2	0.103
Weed vs Red clover	0.290	0.1	0.506
**medium**			
All	0.343	**0.045**	**0.042**
Pairwise comparison			
Control vs Agrochemical	0.234	0.3	0.336
Control vs White clover	0.312	0.1	0.363
Control vs Lupine	0.177	0.6	0.514
Control vs Weed	0.256	0.1	**0.025**
Control vs Red clover	0.245	0.3	0.677
Agrochemical vs White clover	0.196	0.3	0.868
Agrochemical vs Lupine	0.206	0.5	0.928
Agrochemical vs Weed	0.213	0.3	0.071
Agrochemical vs Red clover	0.333	0.1	0.05
White clover vs Lupine	0.237	0.3	0.987
White clover vs Weed	0.203	0.4	0.087
White clover vs Red clover	0.349	0.1	0.097
Lupine vs Weed	0.200	0.5	0.133
Lupine vs Red clover	0.191	0.6	0.307
Weed vs Red clover	0.283	0.1	**0.017**
**Final**			
All	0.316	0.226	0.365
Pairwise comparison			
Control vs Agrochemical	0.189	0.7	0.309
Control vs White clover	0.240	0.1	0.574
Control vs Lupine	0.143	0.8	0.062
Control vs Weed	0.204	0.6	0.217
Control vs Red clover	0.216	0.5	0.332
Agrochemical vs White clover	0.277	0.1	0.251
Agrochemical vs Lupine	0.184	0.8	0.834
Agrochemical vs Weed	0.257	0.2	0.777
Agrochemical vs Red clover	0.200	0.5	0.933

White clover vs Lupine	0.228	0.3	0.128
White clover vs Weed	0.256	0.2	0.24
White clover vs Red clover	0.245	0.3	0.266
Lupine vs Weed	0.191	0.5	0.384
Lupine vs Red clover	0.179	0.7	0.73
Weed vs Red clover	0.173	0.7	0.854
